# Identification and Characterization of a New Microalga *Dysmorphococcus globosus*-HI from the Himalayan Region as a Potential Source of Natural Astaxanthin

**DOI:** 10.3390/biology11060884

**Published:** 2022-06-08

**Authors:** Wafaa F. Zohir, Vikas U. Kapase, Shashi Kumar

**Affiliations:** 1 International Centre for Genetic Engineering and Biotechnology, Aruna Asaf Ali Marg, New Delhi 110067, India; vikas@icgeb.res.in; 2 Botany and Microbiology Department, Faculty of Science, Damanhour University, Damanhour 22511, Egypt

**Keywords:** antioxidant pigments, astaxanthin, freshwater microalgae, DNA barcode, organism identification, phylogenetic analysis

## Abstract

**Simple Summary:**

The natural astaxanthin from algae (marketed only 5%) is the most powerful antioxidant for health compared to the synthetic that shares about 95% of the market. Therefore, there is a huge demand for natural astaxanthin from algae, which can accumulate significantly higher astaxanthin. We have isolated a new algal strain from the Himalayan region, Northern India, which was identified as *Dysmorphococcus globosus*-HI based on morphological and molecular analysis. Its growth conditions were optimized in the laboratory. Among the seven different tested culture media, MBBM and 3N-BBM provided the maximum growth and astaxanthin production. The highest biomass production (1.14 g L^−1^) was observed in the modified BBM medium. It is an excellent source of producing natural astaxanthin, to a tune of 391 mg L^−1^, which is greater than any other known algal species. The productivity of astaxanthin was about 15.6 mg L^−1^ d^−1^ under normal conditions, which is higher than the commercially used *H. pluvialis* species. It is the first report of natural astaxanthin production from *D. globosus*-HI that has great potential for commercial application.

**Abstract:**

Synthesized astaxanthin (ASX), stereoisomers of 3S,3′R, 3R,3′R, and 3S,3′S, have over 95% market share and have relatively poor antioxidant and bioactivity properties, with persistent issues in terms of biological functions, health benefits, and biosafety if compared to natural ASX. Bioprospecting of new microalgal strains could be vital for a new source of powerful antioxidant (ASX). In this study, a new algal strain was isolated from the Indian foothills of the Himalayas. Its identity was discerned by morphological and DNA barcode studies. It is a unicellular spheroidal cell-shaped alga with 100–200 μm diameter. The isolate has 93.4% similarity to *Dysmorphococcus globosus* species based on 18S-rDNA phylogenetic analysis and named as *D. globosus*-HI (HI stands for Himalayan India). Its growth and major cellular components (carotenoids, carbohydrates, protein, lipids, fatty acid profile, and ASX) were optimized using the seven different culture media. The highest biomass (1.14 g L^−1^) was observed in the MBBM medium, with a specific growth rate (0.087 day^−1^), division/day (0.125), and cellular yield (6.16 x 10^6^ cells/mL). The highest carotenoids (1.56 mg g^−1^), lipids (32.5 mg L^−1^), and carbohydrates (135.62 mg L^−1^) were recorded in the 3N-BBM medium. The maximum ω3-FAs (17.78%), ω6-FAs (23.11%), and ω9-FAs (7.06%) were observed in MBBM, JW, and BG-11 medium respectively. The highest amount of antioxidant ASX was accumulated in the 3N-BBM medium (391 mg L^−1^). It is more than any other known algal species used in the production of natural ASX. The optimized biochemical studies on the *D. globosus*-HI strain should fulfill the increasing demand for natural ASX for commercial application.

## 1. Introduction

Microalgae are promising feedstock for biofuels [1,2], cosmetics [3], pharmaceuticals [4], nutrition and food additives [5], and aquaculture [6,7]. Microalgae can be used in agriculture as biostimulants and biofertilizers [8] and bioremediates [9]. They represent a rich biological resource of several valuable pigments, such as carotenoids [10,11]. Carotenoids are associated with several health benefits, i.e., anti-diabetic [12], anti-inflammatory [13], nutraceutical and pharmaceutical applications [14], preventative cardiovascular problems [15], some types of cancer [16], and some diseases of the immunological system [17]. Carotenoids have a high antioxidant capacity [18,19,20], and thus have been reported to protect against premature aging [12,21], UV-radiation [22], and photo-oxidation [14,23,24]. Due to the multiple health benefits of carotenoids, their market demand is rapidly increasing [25,26]. Microalgae associated with carotenoid accumulation could have te potential to meet the increasing market demand for value-added bio-products due to their fast growth, active metabolism, and well-balanced biochemical precursors/pathways [14,27]. Microalgae accumulating secondary carotenoids under environmental stress could be an excellent source of natural carotenoids [19,28]. Exploration of new microalgal strains producing natural carotenoids have been encouraged worldwide for industrial applications [29,30,31].

Among carotenoids, astaxanthin (ASX), a 3,3′-dihydroxylated and 4,4′-diketolated derivative of β-carotene (3,3′-hydroxy-β,β-carotene-4,4′-dione), is one of the most valuable bioproducts due to its high antioxidant capacity [31,32]. The ASX has the most positive antioxidation capacity due to the α-hydroxy ketone functional groups, which involved the singlet oxygen-quenching, reduction of low-density lipoprotein, anticancer activities, and enhancement of immune responses [33,34]. It has been industrially exploited as a feed dye, particularly as a feed supplement in aquaculture and poultry farming [3,6,7]. Due to the exceptional antioxidant capacity of ASX, the trend of its commercial uses in pharmaceutical, nutraceutical, and medical applications is expected to be dramatically increased in the future [25,26].

It is estimated that by 2025 the worldwide global market of ASX shall reach US$ 2.57 billion per year [35]. Currently, a small amount (below 5%) of natural ASX is produced worldwide from freshwater alga *Haematococcus pluvialis* with an approximate cost of US$2500–7000/kg [28,36,37]; the rest about 95% of ASX marketed is chemically synthesized from the petrochemical resources [38,39], comparatively at low cost $1000/kg [40]. However, synthesized ASX has persistent issues in terms of biological functions, health benefits, and biosafety [41]. The synthesized ASX contaminated with other chemical byproducts/intermediates may also have adverse impacts on human health when consumed through supplemented foods and pharmaceuticals [4,38]. Moreover, synthesized ASX has a mixture of (3S,3′R), (3R,3′R), and (3S,3′S) stereoisomers with a proportion ratio of 1:2:1, respectively [26,31], making it an antioxidant with poor properties than natural ones [41]. The natural ASX has two stereoisomers, (3S,3′S)- and (3R,3′R), which are responsible for its greater bioactivity and higher antioxidant properties [26,31,41]. Different biological approaches have been implemented for obtaining ASX from natural resources [38], like direct extraction from marine animals and crustacean waste (krill, shrimp, and crab) [7,33]. The ASX extracted from marine animals/crustacean waste is not suitable for hygienic purposes due to marine pollution, accumulated heavy metals, residual antibiotics, and microplastic contamination in marine animals [11]. Moreover, ASX from marine animals/crustacean waste is low in productivity and includes high extraction costs, thus limiting its commercial-scale production [38]. Despite high production costs, microalgae-based ASX may be advantageous in terms of sustainability, safety control, productivity, and production area [11,38]. 

Besides *Haematococcus pluvialis* (standard ASX-producing algal strains) [12,42,43], other microalgae species are also used to produce a natural ASX, like *Chlorella zofingiensis* [44], *Chlorella protothecoides* [45], *Scenedesmus sp* [46] and *Neochloris wimmeri* [47] with a low amount of ASX (1.1 to 10.72 mg L^−1^). Therefore, there is great need to isolate and identify novel algal strains that can sustain high growth rates and accumulate higher ASX. The ASX productivity is greatly affected by altering the growth conditions, i.e., the culture media [48,49]. Thus, ASX productivity can be improved via media engineering and by thereby improving the growth of an ASX-producing strain. Thus, identifying the best culture medium is vital for attaining the highest ASX yield [19,50]. 

In the present study, we have isolated a new algal strain from the foothills of the Himalayan region, Northern India. This organism gradually turns to orange-red from the green stage during prolonged cultivation, a similar phenomenon is reported for *H. pluvialis*, indicating its importance as a new source of ASX production. The genetic identification and the morphological and biological properties of the isolated microalga were investigated. In addition to the growth characteristics, the composition of the major cellular components, i.e., carbohydrates, protein, lipids, fatty acid profile, and ASX were determined. We aimed to identify the best medium that could be useful in attaining the highest ASX accumulation in the isolated strain. Thus, seven culture media were studied to optimize the strain growth for higher ASX production. The strain was identified as *D. globosus*-HI strain from the Himalayan region, Northern India. It is the first report that highlights its potential for natural ASX production at the par of *H. pluvialis* or more under optimized conditions.

## 2. Materials and Methods

### 2.1. Sample Collection, Isolation, and Growth Conditions

A natural algal strain was isolated from the foothills, Himachal Pradesh, India (latitude 32°6′37.9512″ N and longitude 76°32′10.4064″ E). The collected algal samples were enriched using Bold’s Basal Medium (BBM) (Table S1) by adding ten mL of the collected samples to 50 mL BBM liquid medium in Erlenmeyer flasks (100 mL). A purified culture (axenic culture) was obtained by subjecting the samples to successive rounds of streaking across BBM plates containing antibiotics, i.e., Ampicillin (100 µg mL^−1^) and Cefotaxime (50 µg mL^−1^), and were maintained in axenic conditions by adding one mL of antibiotic into one L of BBM culture medium. The purified algal culture was maintained in the liquid medium, incubated at 25 ± 2 °C, with continuous illumination of light (100 μmolm^−2^ s^−1^), and was kept under constant shaking on an orbital shaker at 150 rpm. 

### 2.2. Molecular Identification and Phylogenetic Analysis

The molecular identification of the isolated strain was conducted using an 18S rDNA sequencing method. To perform the polymerase chain reaction (PCR), total genomic DNA was extracted from 400 mg of wet biomass culture during the exponential phase following the CTAB method [51]. The conserved DNA region corresponding to 18S rDNA was amplified using the 18S primers for the PCR amplification, forward NS1—5′-GTAGTCATATGCTTGTCTC-3′ and reverse NS6—5′-GCATCACAGACCTGTTAT TGCCTC-3′. The PCR amplification was carried out in a 20 μL reaction containing 10 mM dNTP, 5 pmol of each primer, 10× Taq buffer, 0.5 U of Taq polymerase (Real Biotech Corporation, Delhi, India), and 100 ng template DNA. The conditions for PCR amplification were an initial denaturation for 3 min at 95 °C, followed by 35 cycles of denaturation for 30 s at 95 °C, primer annealing at 64 °C for 30 s, and extension for 30 s at 72 °C. The PCR products were separated by Electrophoresis, using 1.5% agarose gel, and visualized in the Bio-Rad Gel doc. system. The size of the amplified DNA “PCR product” using the 18S primers was between 1000 bp and 1500 bp. The DNA sequencing was quantified by NanoDrop (Thermo Fisher one/one ^C^ Microvolume UV-Vis Spectrophotometer) and was carried out by Macrogen Bioservices Inc. Seoul, Korea, and checked for similarity against other publicly available sequences using the Basic Local Alignment Search Tool (BLAST) algorithm to identify homologous taxa available on the NCBI DNA database. The DNA sequences were aligned automatically using the ClustalW alignment algorithm under default parameters using Molecular Genetics Analysis (MEGA X) software [52]. A phylogenetic tree was constructed using the maximum-likelihood algorithms method under default parameters [53] using MEGA X software [54]. The evolutionary distances were computed using the p-distance method [55] and were in the units of the number of base differences per site. Bootstrap resampling analysis was performed to estimate the confidence of the phylogenetic relationship (1000 replicates) [56]. 

### 2.3. Morphological Characterization of Isolate by Light Microscopy

The isolated microalga was examined under a light microscope (LM, Eclipse 80i; Nikon Co., Tokyo, Japan). Images were obtained using a camera (DXM 1200C; Nikon Co., Tokyo, Japan), and the cell size and shape were calculated with an image analyzer (NIS-Elements BR 3.0; Nikon Co., Tokyo, Japan). The cell and shape, chloroplast color and shape, number of pyrenoids, and presence or absence of flagella were recorded.

### 2.4. Assessing Different Growth Media for Optimum Growth

The isolated strain was grown experimentally in 7 common freshwater media (using three replicates), namely BBM [57], MBBM [58], 3N-BBM [59], BG-11 [60], OHM [61], CM Medium, modified [62] and JW (Jaworski’s Medium) [63]. The culture media composition (g L^−1^) and chemical characteristics of the 7 culture media are presented in Table S1. The 7 culture media were individually prepared in autoclaved Erlenmeyer flasks (1 L) with a working volume of 700 mL and reagents of an analytical grade. To avoid nutrient interferences during subculturing, the supernatant of the old medium was discarded, and the starting inoculum was used at the 0.1 OD_750_ for each subculture using a fresh medium. The pH for all the examined media was maintained at 6.9–7.2. The experiment was carried out under a 16:8 light/dark cycle with cool white fluorescent lamps by the side of the flasks providing 100 μmolm^−2^ s^−1^ and constant shaking on an orbital shaker at 150 rpm. When the stock culture reached the early stationary phase (25 days after cultivation), the cells were collected via centrifugation at 6000 rpm for 5 min and biomass (cell dry weight; CDW) and some biochemical parameters were determined as protein and carbohydrates, lipid content, fatty acids, total carotenoids, and ASX content and productivity. 

### 2.5. Growth Characteristics and Biochemical Analysis

#### 2.5.1. Optical Density, Cell Dry Weight (CDW), and Growth Rate

The optical density (OD) of the culture was measured every two days at 750 nm [64] using a UV-1800 Spectrophotometer (Shimadzu, Japan). The dry biomass production (g L^−1^) was quantified twice using dry cell weight and the filtration process. Culture samples (20 mL) were filtered using a pre-weighted Macherey-Nagel GF-1 glass-fiber filter and were dried at 60 °C overnight, then the biomass productivity (g L^−1^ d^−1^) was calculated according to Rizwan et al. [65] using the following Equation (1):

(1) $$\mathrm{Biomass}\;\mathrm{productivity}\;=\frac{X_{f}-X_{i}}{t_{f}-t_{i}}$$

where X_f_ and X_i_ correspond to final and initial CDW (g L^−1^), at the final time CDW (t_f_) and initial time (t_i_), respectively. The initial CDW was measured directly after inoculation of the culture, while the final CDW was after 25 days of cultivation (during the early stationary phase).

The microalgae specific growth rate (μ; d^−1^), doubling time, divisions per day, and maximum cellular yield were estimated according to Guillard [66] and Wood et al. [67] using the following Equations (2)–(5):

(2) $$\mathrm{Specific}\;\mathrm{growth}\;\mathrm{rate}\;\left(\mu \right)=\frac{\;\ln\;\left(\frac{N_{f}}{N_{i}}\right)}{t_{f}-t_{i}}$$

(3) $$\mathrm{Doubling}\;\mathrm{time}\;=\frac{0.6931}{\mu }$$

(4) $$\mathrm{Divisions}\;\mathrm{per}\;\mathrm{day}\;=\frac{\mu }{0.6931}$$

(5) $$\mathrm{Maximum}\;\mathrm{cellular}\;\mathrm{yield}\;=N_{f}-N_{i}$$

where N_f_ and N_i_ correspond to the final and initial cell number (cell mL^−1^), respectively, related to their specific final (t_f_) and initial time (t_i_) in days.

#### 2.5.2. Determination of Chlorophyll *a* and *b* and Total Carotenoid Content

The total chlorophyll *a* and *b* and total carotenoid contents were determined according to Lichtenthaler [68]. One mL aliquot culture was centrifuged, and the pellets were collected while the supernatant was discarded. One mL of methanol (99%) was added to the collected pellets and the samples were stored at 4 °C overnight under dark conditions. Subsequently, samples were centrifuged, and chlorophylls *a* and *b* and carotenoid contents (µg mL^−1^) were estimated by measuring the optical absorbance of the supernatant at 470, 652, and 665 nm using Spectramax M3 multimode spectrophotometer (Molecular Devices, San Jose, CA, USA) and expressed as µg/mL.

(6) Chlorophyll a=16.72 A665.2−9.16 A652.4

(7) Chlorophyll b=34.09 A652.4−15.28 A665.2

(8) $$\mathrm{Total}\;\mathrm{Carotenoids}\;=\frac{\left(1000\;A470-1.63\;\mathrm{Chl}\;a-104.9\;\mathrm{Chl}\;b\right)}{221}$$

#### 2.5.3. Measurement of Protein and Carbohydrates

At the late exponential phase, the total algal biomass was harvested by centrifugation at 6000 rpm for 5 min and dried at 60 °C overnight, then the dried biomass was used for protein and carbohydrates measurements. Protein content was determined following the Lowry method using bovine serum albumin as a standard [69]. Total carbohydrate content was determined following the phenol-sulfuric method and was quantified by a standard glucose curve at 490 nm using Spectramax M3 multimode spectrophotometer (Molecular Devices, San Jose, CA, USA) [70]. Protein and total carbohydrate contents were expressed as mg L^−1^.

#### 2.5.4. Total Lipid Content and Fatty Acids (FAs) Profile Analysis

The total lipids were extracted from the dried algal biomass following the method of Bligh and Dyer [71]. In a glass vial, 500 mg of ground-dried algal biomass (overnight in the oven at 55–60 °C) was mixed with 3 mL of 2:1 chloroform/methanol mixture, then 0.9 mL of distilled water was added and kept overnight on a shaker. After overnight shaking, an additional one mL chloroform and 0.9 mL of distilled water were added, shaken for 4 h and the lipid layers were allowed to separate. The bottom chloroform layer was aspirated and filtered through Whatman No. 1 filter paper into a pre-weighted glass vial. Chloroform was evaporated using a rotary evaporator (BUCHI, Rotavapor II) at 40 °C and the total lipid (mg g^−1^) was calculated by subtracting the pre-weighed glass vial from that of the vial containing the lipid. The lipid productivity (mg g^−1^ day^−1^) was calculated by dividing the lipid content (mg g^−1^) by the number of days “from cultivation to harvest”.

As for the FAs profile analysis, first, the lipid trans-esterification was undertaken by mixing ten mg of the extracted lipids with one mL of hexane (99%) in a test tube, then 200 µL of methanolic KOH (2 M) was used as a catalyst [72] and the mixture was vigorously agitated using a vortexer for 5 min. Then, the upper clear supernatant (hexane) was collected for FAs profile analysis. The quantification of FAs profile analysis was carried out using a gas chromatograph-FID (Agilent GC) equipped with an OmegaWax 250 capillary column (30 m length × 0.25 mm internal diameter × 0.25 µm phase thickness, SUPELCO) and a flame ionization detector. The operating conditions were as follows: a split ratio of 1:10, injection volume of 1 µL, Nitrogen carrier gas with a constant linear velocity of 33.9 cm s^−1,^ and pumping rate of 40-, 400-, and 30-mL min^−1^ for H_2_, air and makeup gas (Nitrogen), respectively. The injector temperature was 270 °C and the detector temperature was 280 °C, whereas the oven temperature started at 140 °C (for 5 min) and then increased at the rate of 4 °C min^−1^ to reach 240 °C, and was then maintained for 20 min. The Heptadecanoic acid (C17:0) was used as an internal standard. Fatty acids were identified by comparing the samples retention times with appropriate FAME standards (Supelco standard FAME mixture-; Sigma Chemical Company, St. Louis, MI, USA) and the data for each individual components are expressed as a percentage of the total content [72].

#### 2.5.5. Astaxanthin Analysis and Quantification

The analytical thin-layer chromatography (TLC analysis) was used for the detection of the pigments and ASX in the isolated algal strain. After successful detection, the HPLC was used for efficient quantification of ASX via their retention values.

##### Thin Layer Chromatography (TLC)

A TLC- pre-coated silica gel H60 thin layer chromatography plate (20 cm × 20 cm Merck, Darmstadt, Germany) was used for the separation of different pigments according to Kobayashi et al. [73] and Chekanov et al. [74] At room temperature, a known weight (15 mg) of the different algal samples was extracted in 2 mL acetone (Sigma-Aldrich Chemical Co., St. Louis, MI, USA), and vortexed with a glass bead for 4 min, centrifuged for 5 min, and this process was repeated until the cell debris became colorless. The algal pigment extracts were dried under N2 gas flow under low light conditions. The samples were resuspended in the petroleum ether, and were then carefully spotted (20 µL of the different samples) to a pre-heated silica Gel chromatography plate, with the Standards (B-carotene and ASX, with a concentration of one gm mL^−1^; Sigma-Aldrich Co., St. Louis, MI, USA) and were allowed to completely dry. The plate was transferred to a highly saturated chamber with a fresh mixture of organic solvents (acetone and n-hexane at a ratio of 30:70% “*v*/*v*”) as a mobile phase and was kept for 30 min at room temperature and under low light conditions. They were then removed and quickly marked on the solvent level and the center of the pigment bands with a pencil. The retardation indices (RFs) for each pigment were calculated using the following equation:

(9) $$\mathrm{RFs}=\frac{\mathrm{Distance}\;\mathrm{traveled}\;\mathrm{by}\;\mathrm{each}\;\mathrm{pigment}\;}{\mathrm{Distance}\;\mathrm{traveled}\;\mathrm{by}\;\mathrm{the}\;\mathrm{solvent}\;\left(\mathrm{mobile}\;\mathrm{phase}\right)}$$

The distance traveled is the distance from the starting point to the geometrical center of the spots corresponding to each pigment or the mobile phase.

##### High-Performance Liquid Chromatography (HPLC)

The ASX was repeatedly extracted (three times) until the cell debris was colorless by adding ten ml of dichloromethane (DCM) to 50 mg of lyophilized dry biomass until the cell debris became colorless. The ASX-DCM extract was evaporated with the HeidolphHei-VAP rotary evaporator, and was then saponified for 3 h at room temperature (23 °C ± 2) under dark conditions by adding 2.25 mL of acetone, 0.25 mL of methanol and 0.5 mL of NaOH (0.05 M in methanol) [75]. Afterward, petroleum ether (three mL) was added, and the mixture was washed with three mL of 10% aqueous NaCl solution, and was then centrifuged for two min at 5500 rpm. The lower phase was discarded and the washing step with the NaCl solution was repeated twice. The organic phase was evaporated and the extracted ASX was dissolved using three mL of solvent B (methanol, methyl tert-butyl ether (MTBE), water, 8:89:3, *v*/*v*) and was filtered with a 0.22 mm disposable nylon syringe filter from Berrytec (Germany). The de-esterified ASX was determined using an HPLC unit (LC-20AB, Shimadzu, Japan) with a diode-array detector (SPD-M20A, Shimadzu, Japan) using a YMC Carotenoid column (C30, 3 mm, 150 × 4.6 mm, YMC Co., Kyoto, Japan). As for the mobile phase, solvent A (methanol, MTBE, water, 81:15:4, *v*/*v*) and solvent B (methanol, MTBE, water, 8:89:3, *v*/*v*) were used with the following gradient: 2% solvent B for 11 min, a linear gradient from 2% solvent B to 40% solvent B for 7 min, 40% solvent B for 6.5 min followed by a linear gradient to 100% solvent B for 2.5 min, 100% solvent B for 3 min, a linear gradient to 2% solvent B for 3 min, held for 7 min. The flow rate was 1 mL min^−1^, the injection volume was 10 µL and the column temperature was maintained at 22 ± 2 °C. For the ASX quantification, a calibration curve was established using standard ASX (Sigma Alderish Company), in which a set of serial dilutions (ranging from 0 to 10 mg L^−1^) of stranded ASX (dissolved in DMSO) were prepared, and then the signal of the diode-array detector was recorded at 478 nm and the corresponding peaks using the HPLC was measured. The limit of detection (LOD) and the limit of quantification (LOQ) were calculated according to the calibration curve [36,76]. The concentration of standard ASX was used as a reference to calculate the concentration of ASX in the sample according to the HPLC peak area. ASX was expressed in mg L^−1^ and as % (*w*/*w*). The ASX productivity (mg L^−1^ day^−1^) was calculated by dividing the ASX content (mg L^−1^) by the number of days, the “cultivation to harvest”. 

The HPLC-grade methanol, MTBE, and ASX were purchased from Sigma-Aldrich Chemical Co. (St. Louis, MI, USA). Other chemicals and reagents were purchased from local companies. Deionized water was purified using a Milli-Q water system (Merck Millipore, Billerica, MA, USA) and was filtered through a 0.22 m^2^ membrane for HPLC analysis.

### 2.6. Statistical Analysis

The obtained data were subjected to ANOVA using one-way CRD in the Glmmix procedure in SAS 9.4 [77]. Differences between means were separated using Tukey’s honestly significant difference HSD test at *p ≤* 0.05. All data were collected in triplicates as mentioned in the figures and tables.

## 3. Results

### 3.1. Cell Morphology 

**Figure 1:**
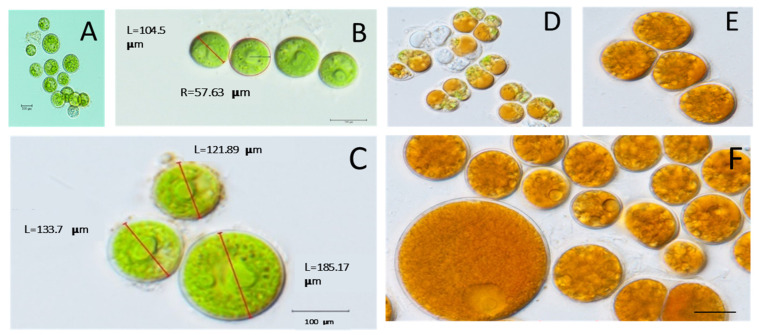
The algal strain isolated from the Himalayan region, rich in astaxanthin (ASX) was identified as *Dysmorphococcus globosus*-HI. Young green culture (young cells) (**A**–**C**) during exponential growth with cells ranging from 100 to 200 μm. The stationary phase of the culture was observed with an orange/red color (**D**–**F**), indicating the accumulation of carotenoid pigments.

**Figure 2:**
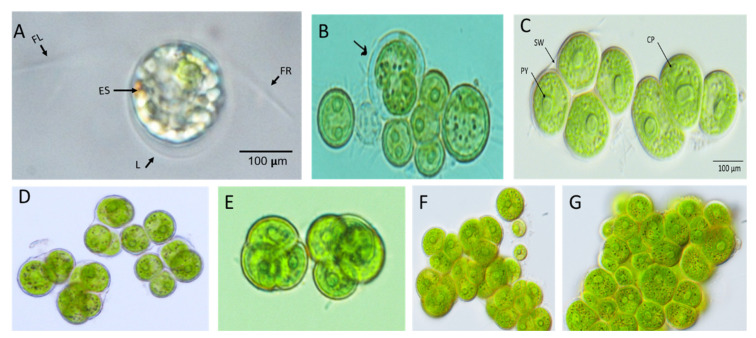
Morphological study of *Dysmorphococcus globosus*-HI cells under the light microscope (100×). It has two isokontic flagella (FL and FR), broadly ovoid-shaped lorica (L) around the green individual cell, and an eye spot (ES) or stigma as a thin or streak-like structure (**A**). Green cells with a 1–2 protruding round pyrenoids (PY) in the center, an urn-like chloroplast (CP), and green cells present in a group are surrounded by a sporangium wall (SW) or the parental lorica (**B**–**G**). Asexual reproduction in *D. globosus*-HI shows the aplanospores that contain 4–16 daughter cells formed per cell (**D**–**G**).

The morphological characteristics of the isolated microalga were investigated using a light microscope. The isolated strain was a unicellular dark green microalga, some cells were observed in aggregation. These cells grew in variable forms/shapes ranging from globose to spheroidal, with a cell size ranging from 100 to 200 μm (Figure 1A,F). Interestingly, the color of the isolated strain changed from green to red, indicating the aging of the culture while accumulating pigments. The growth of *D. globosus*-HI was distinguished based on cells’ age, i.e., young green culture (young cells) (Figure 1A–C), which is characterized by a green color for around 20 days. At the stationary phase, culture without the replacement of a new nutrient medium turned into reddish orange (old cells) (Figure 1D–F). The green and red cells were also observed on agar plates (Figure S1A,B) and in liquid media (Figure S1C,D). Throughout the observations, two ages of the cultures were predominated, and the chloroplast seems to occupy the almost whole-cell lumen (Figure 1A–F). The *D. globosus*-HI cell showed the two isokontic flagella (FL and FR), broadly ovoid-shaped lorica (L) around the green individual cell, and an eye spot (ES) or stigma as a thin or streak-like structure (Figure 2A). The cell wall appears to be hyaline (Figure 2B,C). Each green cell showed 1–2 pyrenoids (PY) surrounded by a starch sheath and has a single urn- or parietal-shaped chloroplast (CP) (Figure 2B,C). Green cells in groups were surrounded by the sporangium wall (SW) or the parental lorica (Figure 2B,C). Evidence for asexual reproduction in *D. globosus*-HI (Figure 2D–G) showed the aplanospores, with each mother cell containing 4–16 daughter cells.

### 3.2. Molecular Identification and Phylogenetic Analysis

In the BLAST analysis, 1121 nucleotides of the 18S rDNA-related gene sequences were obtained from the isolated microalgae. This sequence was assigned an accession number of H210309 after submission to the National Center for Biotechnology Information (NCBI). The nucleotide sequences of the other 16 algal species were obtained from the NCBI database based on the BLAST results of accession number H210309 for comparative alignment analyses of 18S rDNA. The BLAST analysis revealed that our isolate was highly similar to *Dysmorphococcus globosus* as per the NCBI database. The *Dysmorphococcus globosus* SAG 20-1 (KM020136.1) and *D. globosus* (X91629.1) species shared the highest similarities (93.14 to 93.45%) to our isolated microalga.

**Figure 3:**
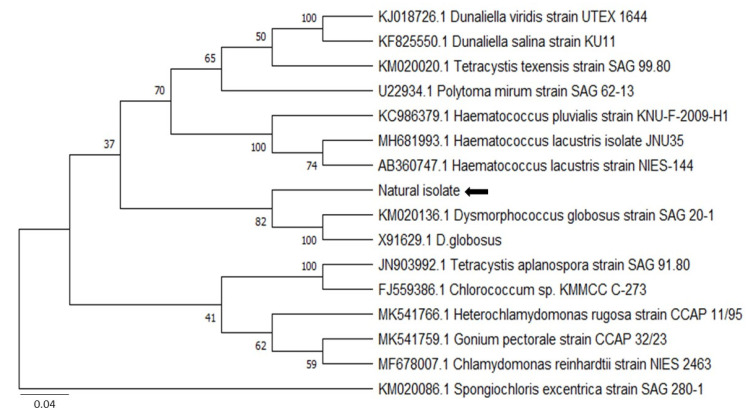
Phylogenetic trees generated using 18S rDNA marker sequences for the isolated microalgal strain. The maximum likelihood method was used to construct the distances within the tree supported by MEGA-X. The branch lengths are proportional to the evolutionary distances. Bootstrap values (>50%) from the bootstrap test (1000 replicates) are shown next to the branches.

The phylogenetic analysis was performed on the basis of blast results (Figure 3) for the maximum likelihood method and involved 16 nucleotide sequences using MEGA X software [54]. All ambiguous positions were removed for each sequence pair (pairwise deletion option) and a total of 2879 positions in the final dataset were observed. The resultant tree using the 18S rDNA genes revealed that the newly isolated microalga strain has a maximum DNA sequence similarity with *Dysmorphococcus globosus* strain SAG 20-1 (KM020136.1) and *D. globosus* (X91629.1) (Figure 3). Based on the phylogenetic analysis, the isolate was taxonomically classified into the genus *Dysmorphococcus*, and was designated *Dysmorphococcus globosus*-HI (Himalayan, India).

### 3.3. Growth Study in Different Media

**Figure 4:**
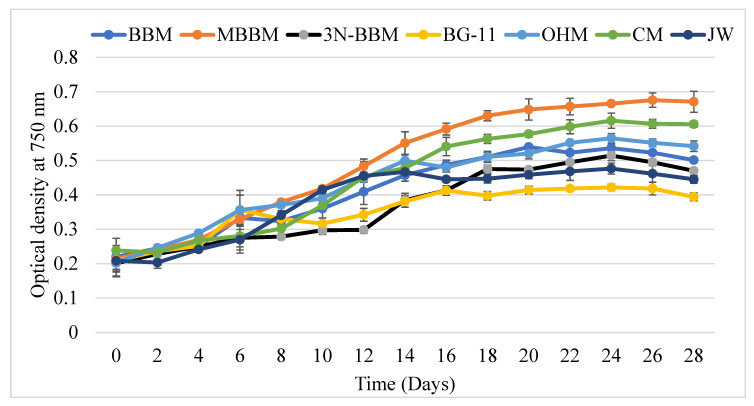
The growth study of *D. globosus*-HI conducted (based on the optical density at 750 nm) in 7 different culture media for 28 days. For details on the culture media, see Table S1. The optical density was measured at an interval of two days.

The isolated algal strain (*D. globosus*-HI) was cultivated under 7 different culture media to identify the best growth medium and improve its production of biochemical components. The growth curve under different culture media of *D. globosus*-HI is presented in Figure 4. The cultures reached a stationary phase in about 22–25 days under the tested media, except in the case of JW where the stationary phase was observed around the 20th day. During the stationary phase, the color of the cells gradually changed from green to yellowish, and thereafter reddish-orange. After day 12, the highest OD was observed in MBBM medium followed by CM and OHM medium. The lowest OD values were observed in BG-11 and JW medium after ~14th day (Figure 4). In the JW medium, the highest growth was observed at the beginning (days 8–14), growth was then stabilized (days 14–20), and after started declining (days 24–28). 

The growth characteristics of the *D. globosus*-HI were significantly (*p ≤* 0.05) affected by the different culture media, the MBBM media (followed by CM and OHM) promoted the growth to the maximum, while BG-11 showed an opposite trend (Table 1). The effect of the different culture media on the growth was observed in the descending order of MBBM > CM > OHM > BBM > 3N-BBM > JW > BG-11 (Table 1). The MBBM media showed significantly higher biomass yield (1.14 g L^−1^), productivity (45.82 mg L^−1^ d^−1^), specific growth rate (0.08 day^−1^), division per day (0.12), and maximum cellular yield (6.16 × 10^6^) compared to the other tested media. In contrast, MBBM, CM, and OHM attained the lowest doubling time (hours). However, the lowest biomass yield (0.56 g L^−1^), productivity (22.60 mg L^−1^ d^−1^), maximum cellular yield (3.18 × 10^6^), specific growth rate (0.06 day^−1^), and division per day (0.08) was recorded in the BG-11 medium with the highest doubling time (12.03 h).

### 3.4. Pigments and Biochemical Composition

#### 3.4.1. Chlorophyll *a*, Chlorophyll *b*, and Total Carotenoids Content

**Figure 5:**
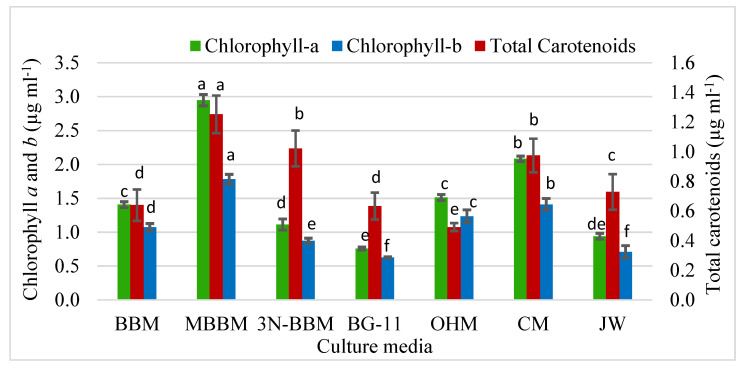
Chlorophyll *a* and *b* and total carotenoids (µg mL^−1^) in *D. globosus*-HI measured on day 25 under photoautotrophic conditions cultivated in 7 different culture media. For the details of media, see Table S1. Bars with similar letters are non-significantly different at *p ≤* 0.05 using Tukey’s test. Letters apply only within the same parameter.

Among all of the 7 tested culture media, chlorophyll *a* was observed higher than chlorophyll *b* and total carotenoids (Figure 5). *D. globosus*-HI had a significantly higher chlorophyll *a* and b content in the MBBM (2.97: 1.78 µg mL^−1^), followed by CM (2.08: 1.41 µg mL^−1^), and OHM (1.51: 1.23 µg mL^−1^), respectively. In contrast, the lowest chlorophyll *a* and *b* contents (0.75: 0.62 µg mL^−1^) respectively were observed in the BG-11 medium. For the total carotenoids, the highest value was observed in MBBM medium (1. 25 µg mL^−1^), followed by 3N-BBM (1.02 µg mL^−1^) and CM (0.97 µg mL^−1^). However, the lowest total carotenoid content was observed in OHM medium (0.50 µg mL^−1^). 

#### 3.4.2. Thin Layer Chromatography of Total Pigment Extracts 

**Figure 6:**
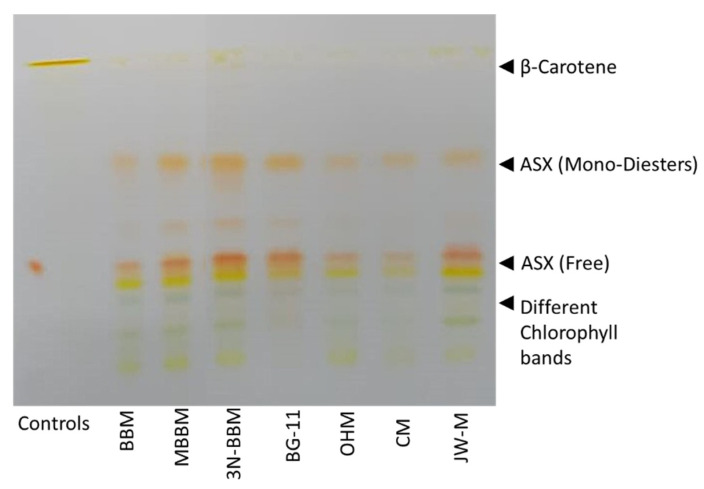
Thin-layer chromatographic analysis of carotenoids in *D. globosus*-HI on day 25 was tested in 7 different culture media. For media composition, see Table S1. The β-carotene was observed as a yellow line on the top of the TLC plate while the remaining mixed bands are a mixture of astaxanthins (ASX) in different forms. The RF values for free-ASX (0.35), ASX diesters and monoesters (0.725 and 0.55), and β-carotene (1.0) were determined. Control standards used were ASX (lower red spot) and β-carotene (upper yellow-orange spot) in the left lane of the TLC plate.

The total pigment extracts of *D. globosus*-HI from different media were spotted on the TLC plates as presented in Figure 6. These were compared with ASX control reference, i.e., the lower band represented the free ASX and β-carotene represented by the upper band. The separation of the different carotenoid pigments on the TLC plate indicated the presence of free ASX, β-carotene, and chlorophylls under different tested media. According to the RFs values, the free ASX showed an RFs value of 0.35, while the astaxanthin monoesters and diesters bands showed RFs values ranging from 0.55 to 0.725 and RFs 1.0 for β-carotene. Below the free ASX bands, several bands ranging from a deep to light green color indicated the presence of pheophytins and chlorophyll pigments.

#### 3.4.3. Proteins and Carbohydrates’ Content

**Figure 7:**
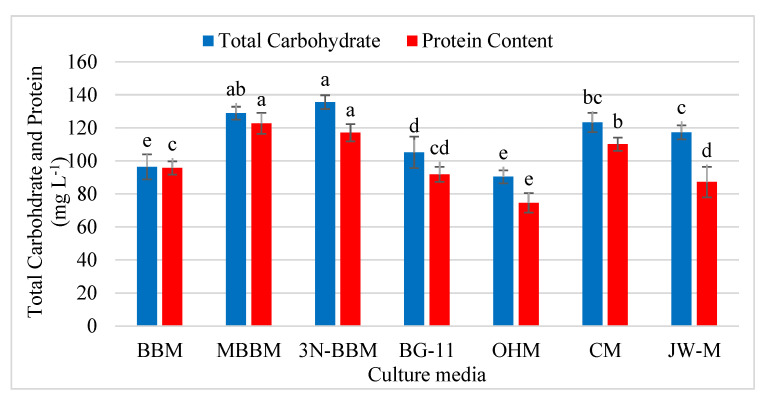
The effect of different culture media on protein and carbohydrate accumulation (mg L ^−1^) in *D. globosus*-HI on 25 days of cultivation. Bars with similar letters are non-significantly different at *p* ≤ 0.05 using Tukey’s test. Letters apply only within the same parameter. For details on media, see Table S1.

The 3N-BBM and MBBM were the most promising media for protein and carbohydrate production (Figure 7). The *D. globosus*-HI accumulated a significantly higher quantity of proteins in MBBM medium (122.78 mg L^−1^ i.e., 10.72% of CDW) on par with 3N-BBM medium (117.13 mg L^−1^ i.e., 15.49% of CDW). The OHM culture medium showed the lowest protein content (74.62 mg L^−1^). Similarly, a significantly higher total carbohydrate yield was observed in 3N-BBM medium (135.62 mg L^−1^), followed by MBBM medium (128.94 mg L^−1^).

The quantity of protein (%) observed in *D. globosus*-HI using the media was as follows, BG-11 (91.92 mg L^−1^; i.e., 18.78% of CDW), 3N-BBM (117.13 mg L^−1^ i.e15.49% of CDW) and JW (87.24 mg L^−1^ i.e., 14.09% of CDW). For carbohydrates, the highest carbohydrate content (%) was attained using the BG-11 medium (105.19 mg L^−1^ i.e., 21.47% of CDW), JW (117.27 mg L^−1^ i.e., 18.92% of CDW) and 3N-BBM (135.62 mg L^−1^ i.e., 17.94% CDW).

### 3.5. Lipid and Fatty Acid Profile Analysis

#### 3.5.1. Lipid Content and Productivity

**Figure 8:**
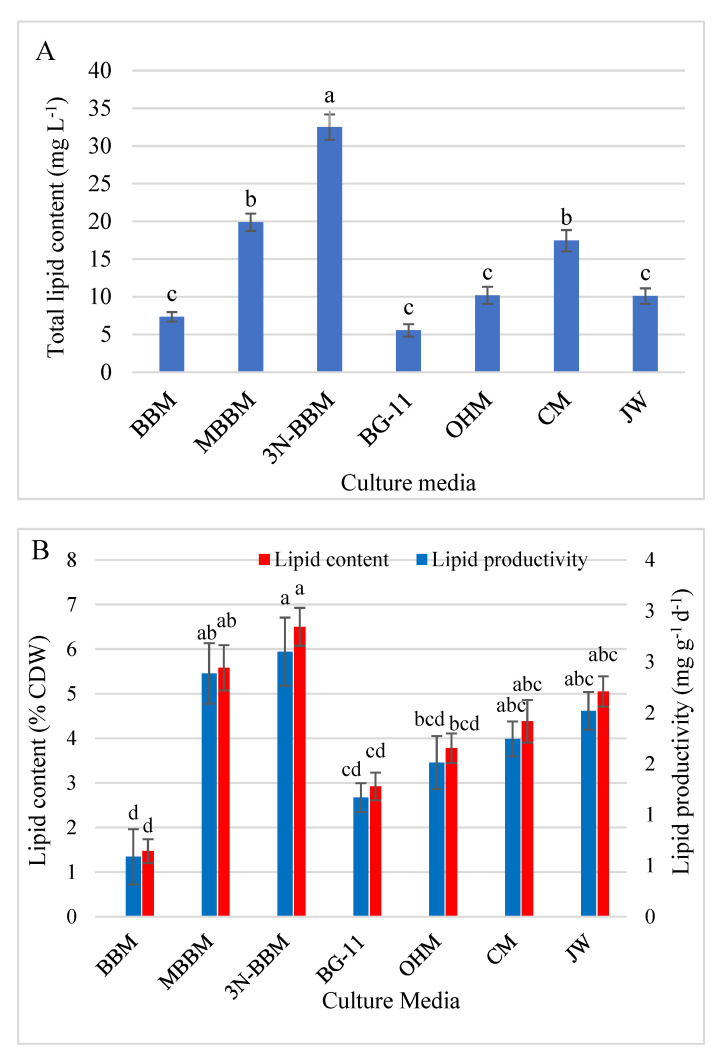
The total lipid content (mg L^−1^) (**A**), lipid percentage (% CDW), and productivity (mg g^−1^ d^−1^) (**B**) in *D. globosus*-HI measured after 25 days of cultivation in the 7 culture media. For the details of the studied media, see Table S1. Bars with similar letters are non-significantly different at *p* ≤ 0.05 using Tukey’s test. Letters apply only within the same parameter.

The total lipid content (mg L^−1^), lipid percentage (% CDW), and lipid productivity (mg g^−1^ d^−1^) for D. globosus-HI were determined under 7 different culture media as presented in Figure 8A,B. Significantly higher total lipid content, lipid percentage, and lipid productivity were recorded for 3N-BBM (32.50 mg L^−1^, 6.50%, and 2.60 mg g^−1^ d^−1^), followed by MBBM medium (19.69 mg L^−1^, 5.58%, and 2.43 mg g^−1^ d^−1^). The lowest total lipid content was observed in BG-11 (5.55 mg L^−1^) and BBM (7.35 mg L^−1^). The lowest lipid percentage and productivity were recorded in BBM (1.47% of CDW, and 0.59 mg g^−1^ d^−1^) and BG-11 (2.92% of CDW, and 1.17 mg g^−1^ d^−1^) respectively.

#### 3.5.2. Profile of Fatty Acids 

**Figure 9:**
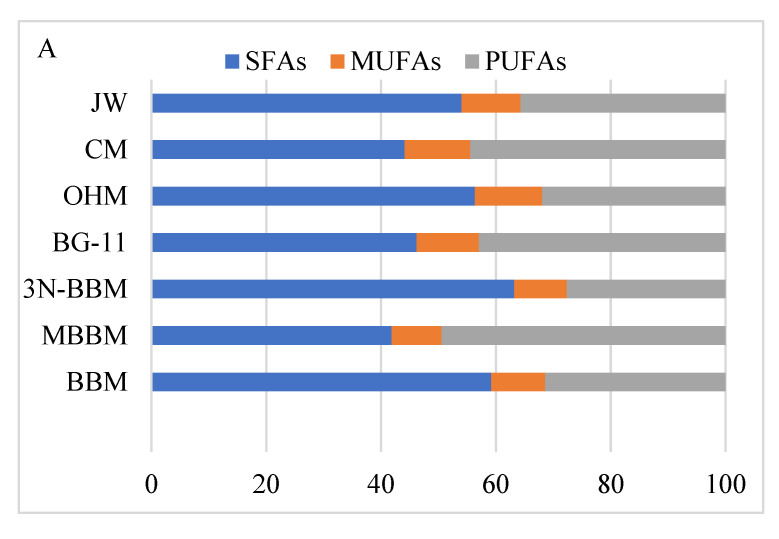

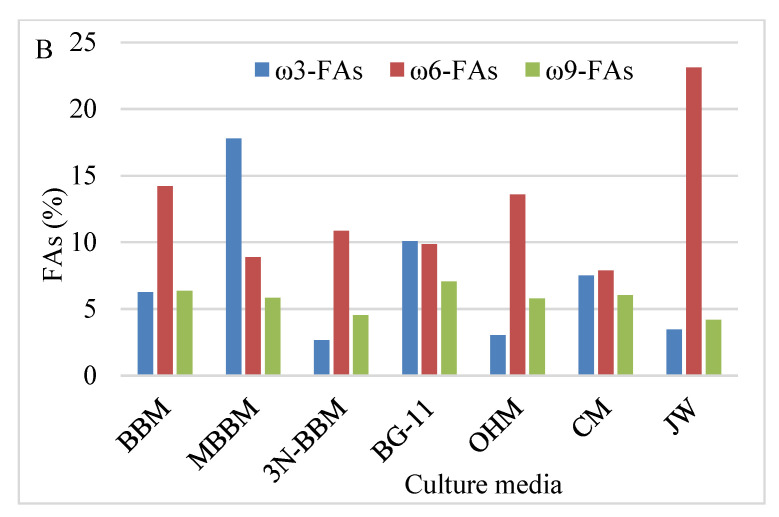
Fatty acids profile analysis of *D. globosus*-HI (25 days of cultivation) in 7 different culture media. For details on media, see Table S1. Percentage of saturated fatty acids (SFAs), monounsaturated fatty acids (MUFAs), and polyunsaturated fatty acids (PUFAs) (**A**). Percentage of Omega 3, 6, and 9 fatty acids (**B**).

The portions of saturated FAs (SFAs), mono- (MUFAs), and poly-unsaturated FAs (PUFAs) present in *D. globosus*-HI after 25 days of cultivation are illustrated in Figure 9A,B. The highest value of SFAs was observed in 3N-BBM (63.20%), BBM (59.19%), OHM (56.36%), and JW (54.06%), whereas MBBM recorded the least total SFAs (41.84%). The effect of the cultivation media on the MUFAs was minimal in the range of 8.71% to 11.70%. The highest total MUFAs was seen in OHM (11.71%), CM (11.49%), BG-11 (10.87%), and JW (10.26%), while the lowest in MBBM (8.72%). The highest value of total PUFAs was observed in MBBM (49.44%), CM (44.43%), BG-11 (42.96%), and JW (35.69%), while the lowest in 3N-BBM (27.64%). Accordingly, the highest total unsaturated FAs were recorded for MBBM (58.06%), CM (55.91%), and BG-11 (53.83%), whereas the lowest were for 3N-BBM (36.81%).

A qualitative and quantitative difference in the FA profile of *D. globosus*-HI was investigated after 25 days of cultivation in 7 different media (Table S2). The 24 FAs (saturated and unsaturated) had 10 to 22 carbon atoms. Irrespective of the used cultivation media, the common SFAs were Caproic acid (C6:0), Behenic acid (C22:0), Caprylic acid (C8:0), and Henicosanoic acid (C21:0); the major MUFAs were Cis-11-Eicosenoic acid (C20:1(n9) and Palmitoleic acid (C16:1); and the major PUFAs were Eicosadienoic acid (C20:2), Eicosatrienoic acid (C20:3n6), Arachidonic acid (ARA) (C20:4n6) and Docosahexaenoic acid (DHA) (C22:6(n3)). 

Caproic acid C6:0 was the dominant SFA (55.11%) with a portion of 34.81% of the total FAs in the 3N-BBM medium (Table S2). The major portion of the MUFAs were 5.96% and 5.06% of Cis-11-Eicosenoic acid (C20:1(n9) and palmitoleic acid (C16:1) in BG11 and BBM, respectively. Similarly, C20:2 was the dominant PUFA (40.50%) with a concentration of 22.63% of total FAs in the CM medium. The data illustrated in Figure 9A,B and Table S3 showed the accumulation of ω3-FAs in MBBM (17.78%), BG-11 (10.10%), and CM (7.51%) respectively, where Docosahexaenoic acid concentration was observed in the range of 47.10 to 81.10%. For ω6-FAs, the highest concentration was observed in JW (23.10%), BBM (14.20%), and OHM (13.59%) respectively. The highest ω9-FAs were found in BG-11 (7.06%), BBM (6.34%), and CM (6.02%) respectively.

### 3.6. Astaxanthin Analysis by HPLC

**Figure 10:**
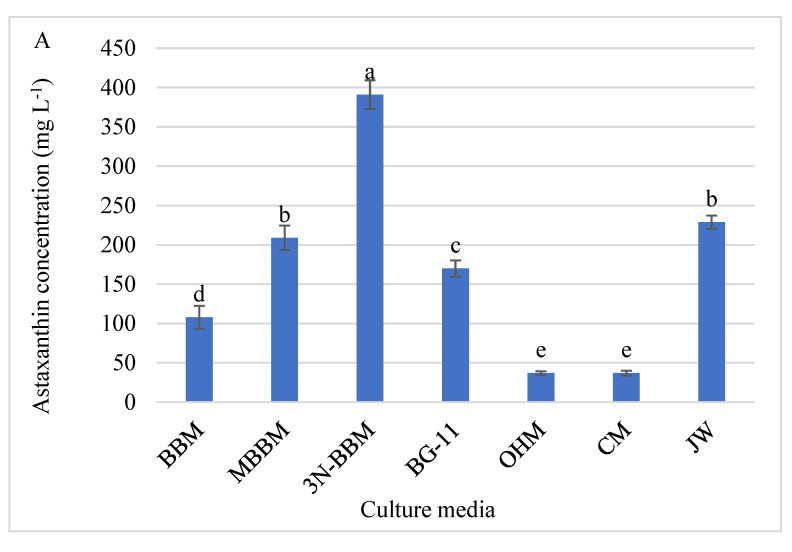

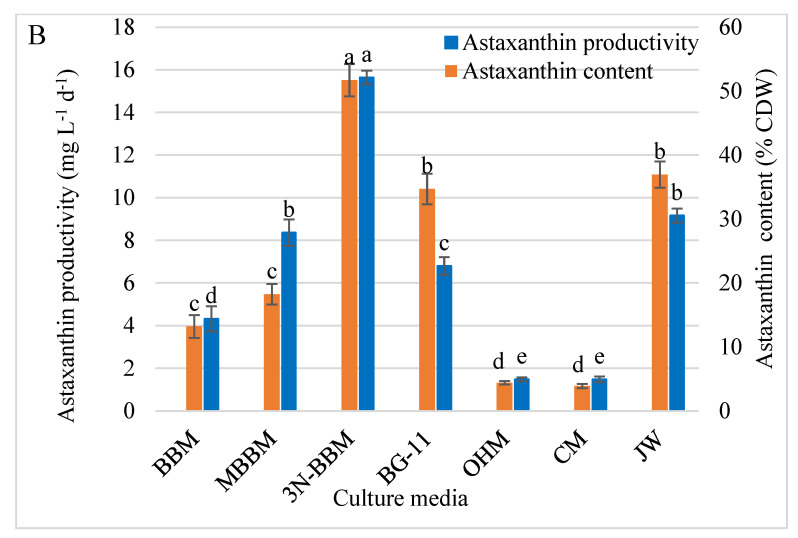
Astaxanthin concentration (mg L^−1^) (**A**); Astaxanthin content (% CDW) and productivity (mg L^−1^ d^−1^) (**B**) were measured for *D*. *globosus*-HI measured after 25 days of cultivation using the 7 different culture media. For media details, see Table S1. Bars with similar letters are non-significantly different at *p* ≤ 0.05 using Tukey’s test. Letters apply only within the same parameter.

The *D. globosus*-HI cells extract showed a peak at the same retention time as the standard ASX, confirming the presence of ASX (Figure S2). ASX was expressed using three different means, (i) ASX concentration (mg L^−1^), (ii) ASX content (% CDW) and (iii) ASX productivity (mg L^−1^ d^−1^) for *D. globosus*-HI tested using 7 different culture media are presented in Figure 10A,B and Table S4. *D. globosus*-HI was able to produce ASX in all media but at different levels. The highest ASX concentration and productivity of *D. globosus*-HI were attained in the 3N-BBM medium, followed by JW and MBBM (Figure 10A,B and Table S4). The lowest ASX concentration was recorded for OHM and CM media. 

As presented in Figure 10B, the ASX content (% CDW) followed a trend that was almost similar to that of ASX concentration (mg L^−1^). The highest ASX content was observed in 3N-BBM (51.71%), JW (36.94%), and BG-11 (34.69%). The smallest ASX content was observed in MBBM (18.25%), BBM (13.21%), OHM (4.39%) and CM (3.89%). The highest ASX productivity (mg L^−1^ d^−1^) was achieved in 3N-BBM, JW, and MBBM media, i.e., 15.64, 9.16, and 8.36 mg L^−1^ d^−1^ respectively. The least ASX productivity was noticed for the MBBM, BG-11, OHM, and CM, i.e., 4.32, 6.80, 4.63, and 1.48 mg L^−1^ d^−1^ respectively. 

## 4. Discussion

### 4.1. Morphological Study

The new isolate has 93.39% 18S rDNA sequence similarity with previously sequenced *Dysmorphococcus sp.* strains (*Dysmorphococcus globosus strain SAG 20-1* (KM020136.1) and *D. globosus* (X91629.1) in the NCBI database; therefore, it was named *Dysmorphococcus globosus*-HI (HI stands for Himalayan India), accession number H210309. The spherical cells of *D. globosus*-HI formed large clusters of aplanospores and dividing cells. Each cell had an urn or parietal-shaped chloroplast, typically 1–2 pyrenoids, with two equal “isokontic” flagella as reported by Bold and Starr [78] for *Dysmorphococcus globosus strain SAG 20-1* and Dawson and Harris [79] for *D. globosus* (X91629.1). The *D. globosus* has a spherical cell up to 28 µm in diameter [78,79]. However, in the present study, the cells of *D. globosus*-HI grown under laboratory conditions were measured 100–200 µm in diameter, which could be due to different geographical and climate conditions and the ability to accumulate more amount of carotenoids.

Regarding asexual reproduction, the cells enlarge, settle to the bottom, and withdraw their flagella. The pyrenoids and nucleus are then divided, followed by the division of the entire protoplast, resulting in four daughters’ cells surrounded by the parental cell wall. These observations of the asexual reproduction for our isolate were similar to those described by Bold and Starr [78], and they also observed 4–16 daughter cells from each mother cell. After the separation of the daughter cells, they grew to the same size as the mother cell and form a spherical, dark aplanospores as mature cells. We have not observed any sexual reproduction during our study as was reported for *Dysmorphococcus* genus [78]. Therefore, we need more focused studies regarding this aspect. Dawson and Harris [79] stated that the sexual reproduction of *Dysmorphococcus* genus is isogamous to anisogamous, with the clones being homothallic.

The genus *Dysmorphococcus* belongs to the family Phacotaceae within the order of the Chlamydomonadales, class Chlorophyceae [80] based on the Algae Base for taxonomy (https://www.algaebase.org/search/species/detail/?species_id=59395) (accessed on 3 April 2022). However, only 5 species of this genus isolated from Asia, Europe, and North America are known, i.e., *D. globosus* [78], *D. variabilis* [80], *D. sarmaii* [81], *D. coccifer* [82], and *D. punctatus* [83]. Among these species, only *D. variabilis* and *D. sarmaii* have been isolated from India. This is the first study that successfully identified the *D. globosus* isolate from India and quantification of its biochemical compounds was estimated.

The change in the color of the culture media (from green to red) indicates the aging of the culture and pigment accumulation. Therefore, the growth of the *D. globosus*-HI was distinguished based on cells’ age, i.e., the green cells were termed young cells, and reddish-orange-colored cells were called old cells. There was a gradual change in the color of the culture media until the entire cytoplasm became orange-red, a phenomenon similarly reported for *H. pluvialis,* known for ASX production. When the orange-red culture was inoculated into a fresh medium, the newly produced flagellated cells were transformed into non-motile cells within 5 days. All these morphological changes are consistent with the typical characteristics of *D. globosus* reported by Dawson and Harris [79] and by Neofotis et al. [84] Thus, based on common findings, this isolate was designated as *D. globosus*-HI, which has potential for astaxanthin production. 

### 4.2. Assessment of an Appropriate Culture Medium 

*D. globosus*-HI was studied in 7 different media in order to identify the best media for the growth and production of a higher amount of biochemical compounds. The growth characteristics of *D. globosus*-HI were studied along with the determination of carbohydrates, protein, lipid, FAs, and ASX contents. The result showed that the isolate could thrive in a broad range of media. However, MBBM medium was observed to be best for supporting its highest growth in terms of maximum OD, CDW, cell numbers, and biomass productivity, whereas BG-11 medium was recorded with the least OD and CDW, cell number, and biomass productivity. The higher biomass under the MBBM medium was consistent with previously published reports for other strains [19,50,85]. These findings might be attributed to the pH buffer system (K_2_HPO_4_-KH_2_PO_4_), a sufficient N supply, and the higher orthophosphate concentration in the MBBM medium compared with other media [50]. The highest final biomass concentration (1.14 gL^−1^) was achieved when cells were harvested on day 25. This result indicated that, compared to several other algal strains, the growth of our isolate could be a challenge, and low biomass production could limit its ability to produce reasonable amounts of bioproducts for commercial requirements. For example, the highest growth obtained under our conditions amounted only to 1.14 g L^−1^, whereas the growth of the well-known ASX source strains (*H. pluvialis*) amounted to 9.00 g L^−1^ in 15 days [19].

### 4.3. Proteins and Carbohydrates Analysis

The highest protein content (18.78% of CDW) was observed for our isolate that was higher than other microalgal strains like *Chlorella vulgaris* (15.67%) [86], *Arthrospira platensis* (13.30%) [87], *Chlorella sorokiniana* (9.90%) [88], *Dunaliella salina* (8.46 %) [89], *Scenedesmus dimorphus* (4.66%) [90], and *Dunaliella tertiolecta* (2.87%) [91]. However, *D. globosus*-HI produced lower amounts of protein than others super protein producing strains, i.e., *Chlamydomonas reinhardtii* (64.70%) [92], *Dunaliella salina* (57.00%) [93], *Porphyridium cruentum* (28–39%) [94], and *Nannochloropsis oceanica* (24.80%) [95]. 

*D. globosus*-HI produced carbohydrate content (21.47% CDW) that was higher than *Scenedesmus obliquus* (13.41 %) [90], *Chlorella vulgaris* (16.00%) [96], and *Tetraselmissuecica*F&M-M33 (NR) (10.32%) [97], but was lower than *Dunaliella salina* (85.58%) [89], *Porphyridium cruentum* (40.01–57.21%) [94], *Tetraselmis suecica* F&M-M33 (S) (36.81%) [97] and *Dunaliella salina* (32.00%) [93]. Thus, based on the biomass yield, our stain may not be a suitable source of protein and carbohydrates. Therefore, enhancing its growth via media engineering could be an essential step to enhance the protein and carbohydrate content.

### 4.4. Lipids and FAs Profile 

Using the *D. globosus*-HI, the highest growth was recorded in the MBBM, and the highest lipid content, lipid percentage, and productivity was recorded in 3N-BBM (32.51 mg L^−1^, 6.50%, 2.60 mg g^−1^ d^−1^) followed by MBBM (19.69 mg L^−1^, 5.58%, 2.43 mg g^−1^ d^−1^). Thus, 3N-BBM medium showed a balanced accumulation of biomass and lipids and attained the highest lipid productivity. It is established that microalgae is a biofuel source, since their lipid content usually ranges from 20 to 50% of the cell dry weight, and can increase to the tune of 80% under specific stress conditions [98,99]. Several algal strains, i.e., *Chlorella* and *Haematococcus*, have been proposed as potential biofuel sources due to their high lipid content, fatty acid composition, and biomass [84,100,101]. However, based on the obtained lipid content and productivity, *D. globosus*-HI stain may not be a potential biofuel source compared to lipid-rich strains. Besides the low lipid content and productivity of the isolate, the FAs profile revealed a high percentage of SFAs (ranging from 42.01–64.12%), indicating poor cold flow properties of the produced biodiesel, because SAFs crystallize at high temperatures and are associated with a high melting point. Therefore crystals are formed at low temperatures [100,102], negatively affecting the quality of the produced biodiesel owing to its unsuitable viscosities and poor cold-flow properties [103].

Conversely, the FAs profile showed that our isolate could be a good source of ω3-FAs when cultivated in MBBM medium (18.01% of the FAs are ω3-FAs), where Docosahexaenoic acid ranged from 47.11 to 81.01%. *D. globosus*-HI could also be used as a source for several PUFAs, i.e., C20:2, C22:6(n3)-ω3, C20:3n6 ω6, C20:4n6-ω6, and cis-11-C20:1(n9)-ω9, that could be used as formula feed for infant nutrition, general feed supplements, and in the pharmaceutical industry [104,105] and industrial petrochemical production [100,106,107]. 

### 4.5. Astaxanthin Analysis

The HPLC analysis revealed that *D. globosus*-HI accumulates the ASX. The *D. globosus* isolated from North America by Neofotis et al. [84], was not quantified for the ASX content or productivity. In the present study, the ASX varied in different culture media from 37.00 to 391.00 mg L^−1^, while the ASX productivity ranged from 1.48 to 15.64 mg L^−1^ d^−1^. Changes in ASX content and productivity due to changes in the growth conditions were reported in other strains [19,100]. For example, Wang et al. [19] revealed that the ASX content varied from 1.60% to 2.70% by changing the growth conditions of *H. pluvialis*-JNU35. Despite the MBBM medium producing the highest biomass (1.14 g L^−1^) than the other tested media, the high biomass came at the cost of the ASX content and productivity, where the highest ASX content (391.00 mg L^−1^) and highest ASX productivity (15.60 mg L^−1^ d^−1^) was attained in the 3N-BBM medium, where the biomass produced only 0.74 g L^−1^. Similar results were obtained by Wang et al. [19] who reported the highest ASX content (2.40%) but not the highest biomass. In the case of 3N-BBM medium, despite a relatively lower growth of *D. globosus*-HI, the highest ASX productivity was recorded (in 3N-BBM medium) due to the high ASX content, confirming that the high ASX content is vital for attaining the highest ASX productivity. The *H. pluvialis* (standard ASX source) can accumulate lipids under stress conditions, which increases the cell size and improves ASX content, as lipids can serve as a supportive matrix, allowing for ASX pending in the cytoplasm [19,32,108]. Several authors revealed that β- carotene converts to ASX in lipid bodies and ASX accumulation is proportional to lipid synthesis [109,110,111]. Our results supported this concept through 3N-BBM medium, which showed the highest ASX content and productivity, along with the highest lipid content and productivity. Moreover, for the 7 tested media, a strong positive correlation has been observed between lipids- and ASX productivity in which the r value was 0.94, R^2^ was 0.88 and the correlation has been described by a third-degree a polynomial equation (Figure S3). Chen et al. [112] studied the correlation between ASX- and lipids accumulation and reported that an ASX esterification step might be responsible for this correlation. The authors indicated that increasing the titer of lipid content might be able to increase the ASX and that overexpression of relevant esterifying enzymes might be able to promote both lipid and ASX synthesis [112]. Under stress conditions (N-starvation), ASX-producing strains, i.e., *Haematococcus*, could divert C-partition from starch to lipids and ASX via increased activity of the tricarboxylic acid cycle [113]. The ASX accumulation could be induced by nutrient deficiency, especially N-deprivation. One evidence is that the cells reach the stationary phase (cells became orange-red indicating ASX accumulation) without the replacement of a new nutrient medium and when orange-red cells were inoculated into a fresh medium, the newly produced cells completely turn green within 5 days. 

In our study, the highest ASX content (391.00 mg L^−1^) observed in *D. globosus*-HI was higher than any known potential algal ASX source, where the genus *Haematococcus* has an ASX content ranging from 7.72 to 174.70 mg L^−1^ [19,50,85,100,114]. Similarly, the ASX percentage (CDW basis) in our isolate (51.01% CDW) was also higher than any observed values for *Haematococcus* (1.00 to 5.00%) and *Bracteacoccus aggregatus* BM5/15 (48.01%) [74]. More importantly, despite the low biomass of *D. globosus*-HI, ASX productivity (15.00 mg L^−1^ d^−1^) was promising, which is higher than any other algal strains [50,85,100,114]. The only exception is *H*. *pluvialis* JNU35, only when cultivated at a photobioreactor scale under specific stress conditions (different N-sources and light conditions) [19]. 

Our results indicate that *D. globosus*-HI has excellent genetic properties with a higher accumulation of ASX. *D. globosus*-HI could provide a comparable ASX productivity to *Heamatococcus sp.*, despite its low biomass productivity. As observed for *Heamatococcus* sp. [19,32,50,115], subjecting *D. globosus*-HI to specific growth conditions, i.e., nutrient deprivation/enrichment, salinity, irradiance, and temperature, could further enhance its growth, ASX content, and productivity. The *D. globosus*-HI strain produced low biomass; therefore, it requires further media engineering to enhance its growth. In particular, C-enrichment could be a win–win solution that might provide high growth without decreasing the ASX accumulation. Several authors revealed that a higher biomass and ASX yield could be achieved by altering the C/N ratio through C-enrichment [109,116,117,118]. In our study, ASX was determined during the early stationary phase; however, ASX content and productivity could differ if measured during the mid- and/or the late stationary phase. Future research is required to determine the best harvesting time to get the highest ASX productivity. Therefore, in the future, work should be focused on enhancing the growth of *D. globosus*-HI without affecting its ASX content through the optimization of physio-chemical environmental conditions (C-enrichment and illumination) and/or genetic modification. 

## 5. Conclusions

A new microalgal strain was successfully isolated from the Himalayan region, Northern India. Based on the morphological and molecular analysis, the isolated strain was identified as *D. globosus*-HI. This is the first report that quantifies its potential to produce value-added bioproducts and highlights its great potential as a new source of natural ASX. Among the 7 culture media, MBBM and 3N-BBM were the best media that boosted the growth and biochemical production in the strain. Interestingly, *D. globosus*-HI could be an exceptional source for the natural ASX in which the ASX content amounted to the tune of 391.00 mg L^−1^, which is greater than any known ASX source from algae. The ASX productivity amounted to 15.60 mg L^−1^ d^−1^ in the 3N-BBM, which is also higher than the other algal strains. Further research is required to enhance *D. globosus*-HI growth and improve its ASX content through the optimization of physio-chemical environmental conditions and/or genetic modification.

## Figures and Tables

**Table 1:** The effect of different culture media on growth parameters like cell dry weight (CDW), biomass productivity, growth rate, division per day, maximum cell number, and doubling time of the isolated algal strain (*D. globosus*-HI).

Culture Media	Cell Dry Weight (g L^−1^)	Biomass Productivity (mg L^−1^ d^−1^)	Specific Growth Rate (day^−1^)	Division Per Day (K)	Maximum Cellular Yield (×10^6^ cells mL^−1^) (R)	Doubling Time (h)
BBM	0.817 ± 0.014c	32.680 ± 0.545c	0.065 ± 0.005d	0.093 ± 0.011c	3.600 ± 0.314d	10.720 ± 0.020b
MBBM	1.145 ± 0.009a	45.820 ± 0.364a	0.087 ± 0.002a	0.125 ± 0.022a	6.160 ± 0.294a	7.994 ± 0.100e
3N-BBM	0.756 ± 0.051d	30.240 ± 0.203d	0.059 ± 0.003e	0.085 ± 0.002d	3.050 ± 0.483e	11.773 ± 0.140a
BG-11	0.565 ± 0.011f	22.600 ± 0.454f	0.058 ± 0.009e	0.083 ± 0.003	3.180 ± 0.108e	12.032 ± 0.010a
OHM	0.846 ± 0.006c	33.440 ± 0.294c	0.070 ± 0.006c	0.101 ± 0.005b	4.460 ± 0.312c	9.901 ± 0.070c
CM	0.950 ± 0.001b	38.000 ± 0.605b	0.081 ± 0.004b	0.116 ± 0.040b	5.300 ± 0.424b	8.590 ± 0.090d
JW	0.620 ± 0.007e	24.800 ± 0.263e	0.060 ± 0.009e	0.086 ± 0.003d	3.34 ± 0.122e	11.601 ± 0.20a

Algal samples were harvested on day 25 of the cultivation. Values are the mean ± SD (n = 3). Values with similar letters are non-significantly different at *p ≤* 0.05 using Tukey’s test. Letters apply only within the same parameter. For the details of the used media, see Table S1.

## Data Availability

Data are contained within the article.

## Supplementary Materials

The following supporting information can be downloaded at: https://www.mdpi.com/article/10.3390/biology11060884/s1, Table S1: Culture media composition (g L^−1^) and chemical characteristics; Table S2: The fatty acid profile of *D. globosus*-HI measured after 25 days of cultivation in the 7 culture media and relative percentages of each fatty acid. For media details refer to Table S1; Table S3: The percentage of different Omega 3, 6 and 9 fatty acids (% of total FAs) in *D. globosus*-HI measured after 25 days of cultivation for the 7 culture media; Table S4: Astaxanthin concentration (mg L^−1^), total dry weight (g L^−1^), and astaxanthin productivity (mg L^−1^ d−^1^) of *D. globosus*-HI measured after 25 days of cultivation for the 7-culture media (for details on media composition see Table S1); Figure S1: The green and red stages of culture observed on an agar plates (A and B) and in liquid media (Figure S1 C and D); Figure S2: The HPLC peak area of the standard Astaxanthin and Astaxanthin in *D. globosus*-HI extractes harvested after 25 days of cultivation for the 7 culture media, confirming the presence of ASX in the *D. globosus*-HI. For details on the studied culture media composition, see Table S1; Figure S3: A third degree a polynomial correlation between lipids- and ASX productivity under 7 culture media. The r value is 0.94, R^2^ was 0.88.
