# Supplementary material for: Identification and Characterization of a New Microalga Dysmorphococcus globosus-HI from the Himalayan Region as a Potential Source of Natural Astaxanthin
Source: Biology (Basel). 2022 Jun 8;11(6):884. doi: 10.3390/biology11060884 (PMC9220219; doi:10.3390/biology11060884)
Supplement: Supplementary file 1 [file biology-11-00884-s001.zip › biology-1721402-supplementary.pdf]

Supplementary Materials

Supplementary Tables

Table S1. Culture media composition studied for *D. globosus*-HI.

Components	BBM	MBBM	3N-BBM	BG-11	OHM	CM	JW
Tris base						0.50	
KNO_3					40.00	0.10	
$NaNO_3$	25.00	250.00	75.00	1.50			0.08
$MgSO_4 \cdot 7H_2O$	7.50	75.00	7.50	75.00	24.50	40.00	0.05
$CaCl_2.2H_2O$	2.50	25.00	2.50	36.00	11.00		
K_2HPO_4	7.50	75.00	7.50	40.00			
$NaHCO_3$							0.016
KH_2PO_4	17.50	1.75	17.500				0.012
Na_2HPO_4					30.00		0.036
$Na_2EDTA.2H_2O$		10.00		1.00		1.00	0.002
EDTAFeNa							0.002
KOH	31.00	6.20	31.00				
EDTA	50.00		50.00				
$FeCl_3 \cdot 6H_2O$						0.194	
NaCl	2.500	25.00	2.500				
Ca $(NO_3)_2.4H_2O$						0.150	0.020
Na-B-glycerophosphate. $5H_2O$						50.00	
Citric Acid$\cdot H_2O$				6.00			
Ferric Ammonium Citrate				6.00			
Ferric III Citrate					2.62		
$FeSO_4 \cdot 7H_2O$	4.98	4.98					
H_2SO_4 (ml)	1.00	1.00	1.00				
Cr_2O_3					0.075		
SeO_2					0.005		
Na_2CO_3				20.00			
H_3BO_3	11.42	11.50-2.86	11.42	2.86			0.0025
$ZnSO_4 \cdot 7H_2O$	8.82	0.222	8.82	0.22			
$ZnCl_2$						10.440	
$CoCl_2 \cdot 6H_2O$					0.011	4.040	
$CuSO_4 \cdot 5H_2O$	1.57	0.079	1.57	79.00	0.012		
Co $(NO_3)_2 \cdot 6H_2O$	0.49	0.049	0.49	49.40			
$MnCl_2 \cdot 4H_2O$	1.44	1.81	1.44	1.810	0.908	36.00	0.0014
MoO_3	0.71		0.71				
$Na_2MoO4 \cdot 2H_2O$		0.39		0.391	0.12	12.62	
$(NH4)_6Mo_7O_{24} \cdot 4H_2O$							0.001
Thiamine		0.20			17.5*		40.0*
Cyanocobalamin (B_{12})		0.001			15.0*	0.1	40.0*
Biotin		0.001			25.0*	0.1	40.0*
pH	6.60	6.80	5.50	7.10	7.00	7.50	6.90

All media compsotion are used in (g L^{-1}) except (μg L^{-1})*.

Table S2. The fatty acid profile of *D. globosus*-HI measured after 25 days of cultivation using seven different culture media. For media details, refer to Table S1.

Fatty Acid (Relative Percentage)								
Saturated fatty acids (SFAs)								
	Symbols	BBM*	MBBM	3N-BBM	BG-11	OHM	CM	JW
Caproic acid	C6:0	24.479	19.765	34.811	14.089	22.062	10.362	19.664
Caprylic acid	C8:0	5.813	3.573	5.324	4.848	6.794	6.782	6.943

Capric acid	C10:0	1.573	1.011	1.160	1.348	1.919	1.463	1.763
Undecanoic acid	C11:0	1.492	1.553	2.424	1.130	2.212	2.395	2.351
Myristic acid	C14:0	3.140	1.958	2.232	2.746	3.919	3.173	3.538
Stearic acid	C18:0	1.313	0.779	0.870	1.105	1.531	1.069	1.500
Arachidic acid	C20:0	3.215	3.539	3.070	3.404	3.131	3.295	3.144
Henicosanoic acid	C21:0	6.322	0.973	5.113	8.199	5.758	6.718	5.744
Behenic acid	C22:0	7.676	8.688	5.457	9.304	5.977	7.270	6.131
Tricosanoic acid	C23:0	4.161	0.000	2.736	0.000	3.057	1.560	3.284
**Total SFAS**		59.185	41.839	63.197	46.172	56.359	44.087	54.062
Unsaturated Fatty Acids (USFAs)								
**Mono-UFAs**	Symbols	BBM	MBBM	3N-BBM	BG-11	OHM	CM	JW
Myristoleic acid	C14:1	0.000	1.057	2.565	1.224	2.303	2.529	2.755
Palmitoleic acid	C16:1	3.037	1.831	2.064	2.578	3.640	2.935	3.324
Oleic acid (ω9)	C18:1 cis(n9)	1.324	0.766	0.873	1.105	1.549	1.111	0.000
Cis-11-Eicosenoic acid	C20:1(n9)	5.025	5.066	3.657	5.963	4.220	4.912	4.180
**Total MUFAs**		9.386	8.721	9.159	10.871	11.712	11.486	10.259
**Poly-UFAs**	**Symbols**	**BBM**	**MBBM**	**3N-BBM**	**BG-11**	**OHM**	**CM**	**JW**
Linolelaidic acid	C18:2 trans (n6)	0.880	1.510	1.260	1.720	1.980	1.81	1.890
Linoleic acid	C18:2 cis (n6)	0.910	1.140	0.570	0.740	1.060	0.7000	1.000
γ-Linolenic acid	C18:3n6	0.000	0.000	0.440	0.580	0.840	0.570	0.820
α-Linolenic acid	C18:3n3	0.000	7.660	0.000	0.000	0.000	0.000	0.000
cis-11,14-Eicosadienoic acid	C20:2	10.970	15.090	14.150	14.720	15.300	22.630	18.420
cis-8,11,14-Eicosatrienoic acid	C20:3n6	6.910	0.000	4.790	0.000	5.370	0.000	5.570
Arachidonic acid	C20:4n6 (ARA)	5.500	6.230	3.790	6.810	4.350	4.810	4.540
cis-5,8,11,14,17-Eicosapentaenoic	C20:5n3 (EPA)	1.460	1.730	0.930	1.870	1.050	1.590	1.250
cis-13,16-Docosadienoic acid	C22:2	0.000	7.690	0.000	8.300	0.000	6.390	0.000

cis-4,7,10,13,16,19-Docosahexaenoic acid	C22:6(n3) (DHA)	4.790	8.390	1.710	8.220	1.980	5.920	2.190
**Total Poly-USFs**		31.430	49.440	27.640	42.960	31.930	44.430	35.680
**Total USFs**		40.810	58.060	36.800	53.830	43.6420	55.9130	45.930

SFAs, Saturated fatty acids; UFAs, Unsaturated fatty acids; MUFAs, Monounsaturated fatty acids; PUFAs, Polyunsaturated fatty acids.

Table S3. Percentage of different Omega 3, 6 and 9 fatty acids in *D. globosus*-HI (% of total FAs) measured on day 25 cultivated in seven different culture media. For medium details refer to Table S1.

	**ω3**	**Carbon atoms**	**BBM**	**MBBM**	**3N-BBM**	**BG-11**	**OHM**	**CM**	**JW**
**Omega 3**	α-Linolenic acid	C18:3n3	0.000	7.663	0.000	0.000	0.000	0.000	0.000
	Eicosapentaenoic	C20:5n3 (EPA)	1.459	1.731	0.934	1.870	1.046	1.594	1.247
	Docosahexaenoic acid	C22:6(n3) (DHA)	4.792	8.393	1.710	8.222	1.981	5.920	2.194
	Total Omega 3		6.252	17.788	2.645	10.091	3.027	7.514	3.441
	**ω6**	**Carbon atoms**	**BBM**	**MBBM**	**3N-BBM**	**BG-11**	**OHM**	**CM**	**JW**
**Omega 6**	Eicosatrienoic acid	C20:3n6	6.908	0.000	4.794	0.000	5.368	0.000	5.568
	γ-Linolenic acid	C18:3n6	0.000	0.000	0.442	0.585	0.836	0.566	4.541
	Arachidonic acid	C20:4n6 (ARA)	5.498	6.227	3.786	6.813	4.353	4.808	10.109
	Linolelaidic acid	C18:2 trans (n6)	0.885	1.512	1.261	1.718	1.984	1.809	1.893
	Linoleic acid	C18:2 cis (n6)	0.913	1.140	0.571	0.735	1.058	0.703	0.995
	Total Omega 6		14.204	8.879	10.854	9.851	13.598	7.886	23.107
	**ω9**	**Carbon atoms**	**BBM**	**MBBM**	**3N-BBM**	**BG-11**	**OHM**	**CM**	**JW**
Omega 9	Oleic acid ω9	C18:1 cis(n9)	1.324	0.766	0.873	1.105	1.549	1.111	0.000
	cis-11-Eicosenoic acid	C20:1(n9)	5.025	5.066	3.657	5.963	4.220	4.912	4.180
	Total Omega 9		6.349	5.832	4.530	7.068	5.769	6.023	4.180

Table S4. Astaxanthin concentration (mg L^{-1}), total dry weight (g L^{-1}), and astaxanthin productivity (mg L^{-1} d^{-1}) of *D. globosus*-HI measured on day 25 cultivated in seven differnt culture media. For media composition, see Table S1.

**Media**	**Astaxanthin concentration (mg L^{-1})**	**Cell dry weight (g L^{-1})**	Astaxanthin content mg g^{-1}	Astaxanthin content (%)	Astaxanthin productiv-ity (mg L^{-1} d^{-1})
**BBM**	**108.000±14.697 d**	**0.817± 0.014 c**	132.130±17.852 c	13.213±1.785 c	4.320±0.588 d
MBBM	209.000 ±15.513 b	1.145± 0.009 a	182.450±16.158 c	18.245±1.616 c	8.360±0.621 b
3N-BBM	391.000±8.179 a	0.756±0.051d	517.090±5.266 a	51.709±2.527 a	15.640±0.327 a
BG-11	170.000±10.274 c	0.490±± 0.011f	346.940±23.900 b	34.694±2.390 b	6.800±0.411 c
OHM	37.000±2.449 e	0.846±0.006 c	43.740±2.700 d	4.394±0.270 d	1.480±0.098 e
CM	37.000±3.266 e	0.950±0.001b	38.950±3.219 d	3.893±0.322 d	1.480±0.131 e
JW	229.000±8.219 b	0.620±0.007e	369.350±20.540 b	36.935±2.054 b	9.160±0.329 b

Supplimentary Figures

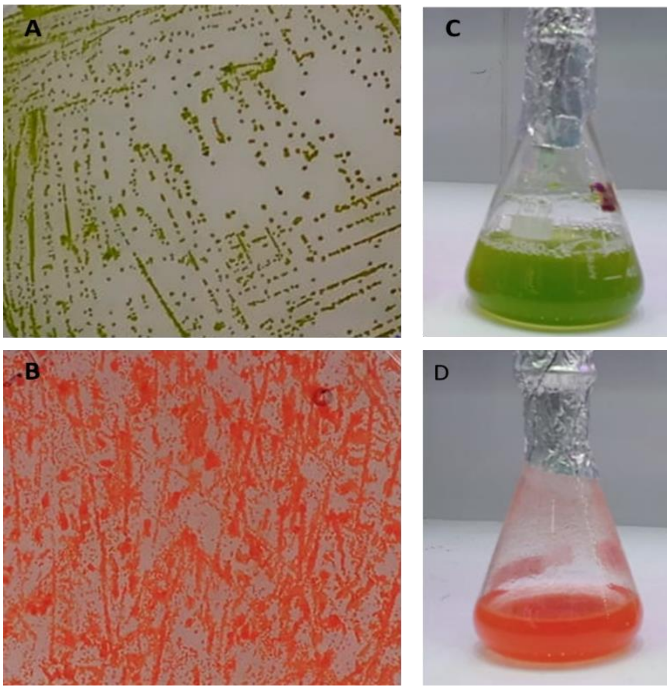

Figure S1. The green and red stages of culture observed on an agar plates (A-B) and in liquid medium (C-D).

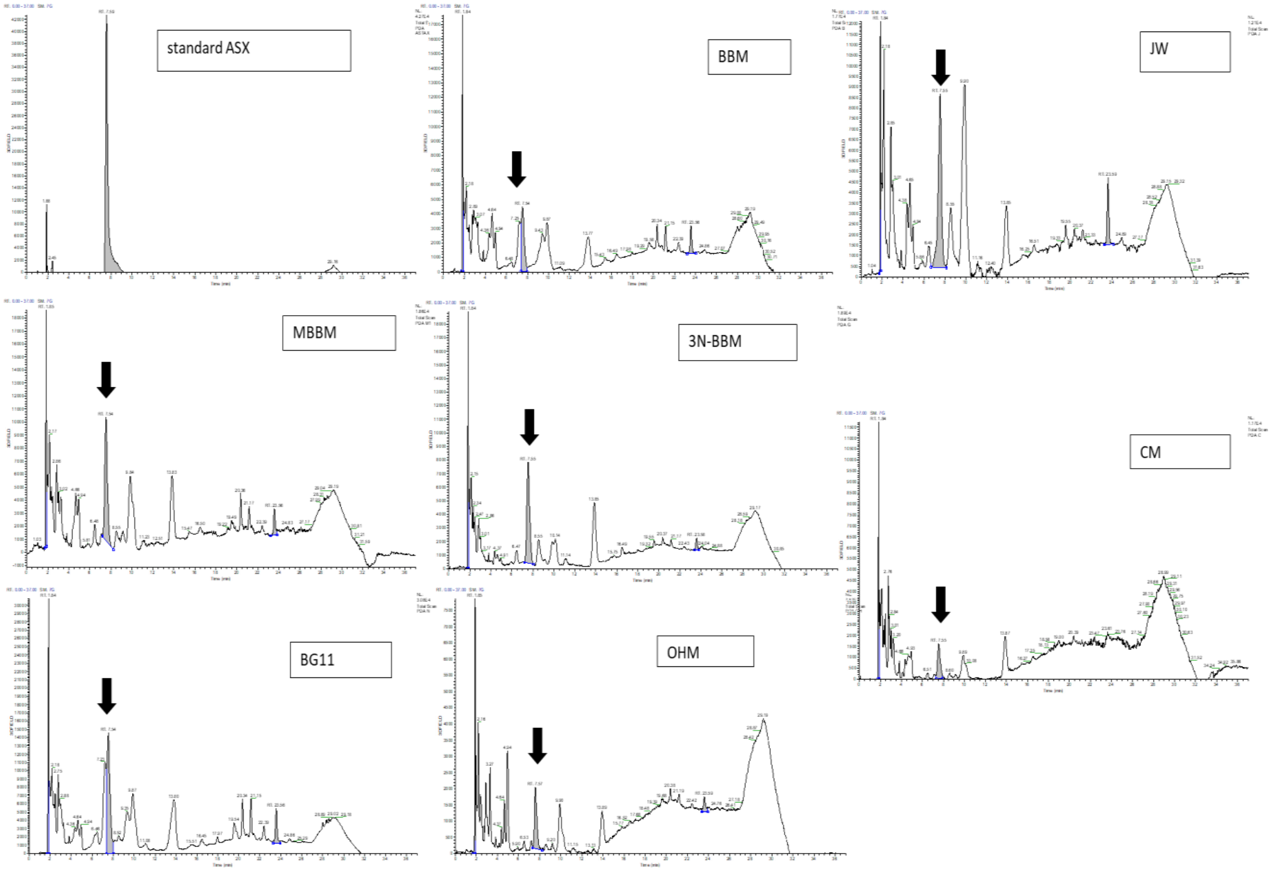

Figure S2. The HPLC peak area of the Standard Astaxanthin and Astaxanthin observed in *D. globosus*-HI extracts, harvested on day 25 of cultivation in differnt culture media. The presence of ASX in D. globosus-HI isolate is denotaed by dark arrow. For details of culture media tested, see Table S1.

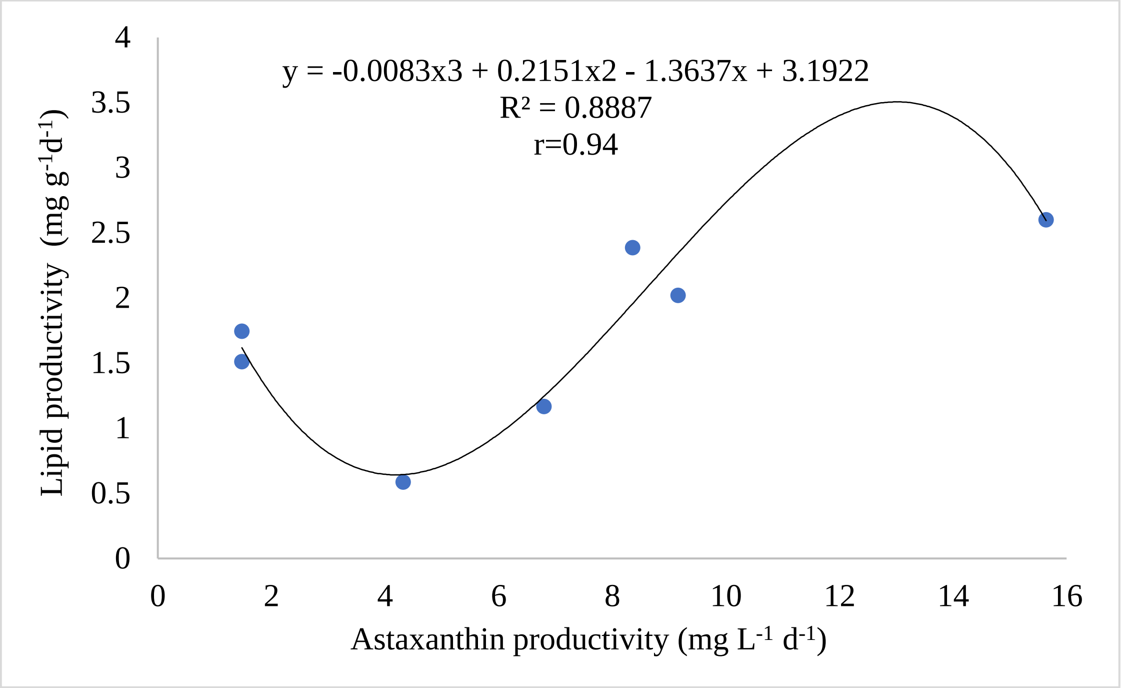

Figure S3. A third degree a polynomial correlation between lipids- and ASX productivity using seven differnt culture media. The **r** value is 0.94, $\mathbf{R^2}$ is 0.88.
